# Burden of illness for patients with primary biliary cholangitis: an observational study of clinical characteristics and healthcare resource utilization

**DOI:** 10.57264/cer-2024-0174

**Published:** 2025-03-06

**Authors:** Robert G Gish, Joanna P MacEwan, Alina Levine, Dannielle Lebovitch, Leona Bessonova, Darren Wheeler, Radhika Nair, Alan Bonder

**Affiliations:** 1 Robert G Gish Consultants, LLC, San Diego, CA 92037, USA; 2 Genesis Research Group, Hoboken, NJ 07030, USA; 3 Intercept Pharmaceuticals, Morristown, NJ 07960, USA; 4 Division of Gastroenterology & Hepatology, Beth Israel Deaconess Medical Center, Harvard Medical School, Boston, MA 02215, USA

**Keywords:** acute care, healthcare resource utilization, liver cirrhosis, primary biliary cholangitis

## Abstract

**Aim:**

To evaluate the clinical characteristics and healthcare resource utilization for acute care and its costs for patients with primary biliary cholangitis (PBC) with or without cirrhosis.

**Materials & methods:**

This retrospective observational cohort study was conducted using two datasets (Komodo’s Healthcare Map™ [Komodo Health] and Optum Clinformatics^®^ Data Mart [CDM] database) between 2015 and 2023. Patients (≥18 years) with PBC were identified based on ≥1 inpatient or ≥2 outpatient claims. Healthcare resource utilization for acute care (hospitalizations and emergency department [ED] visits [not leading to hospitalization]) were assessed in both datasets, and associated medical costs were evaluated in Optum CDM.

**Results:**

In Komodo Health, of the 29,758 patients with PBC (mean age: 59.2 years), 21.6% had cirrhosis and 50.4% of patients with cirrhosis had Medicaid or Medicare coverage. Of the total 8143 patients in Optum CDM (mean age: 67.0 years), 20.7% had cirrhosis, and most were enrolled in Medicare (69.7%). There was a larger proportion of men in the cirrhosis group compared with the no-cirrhosis group in Komodo Health (31.7 vs 16.3%) and Optum CDM (29.7 vs 16.5%). Annually, among patients with cirrhosis who had a hospitalization, 69.3% had additional hospitalizations, and among patients who had an ED visit, 52.9% had additional ED visits in Komodo Health; similar results were observed in Optum CDM. Among patients with at least one acute-care event, the mean annual acute-care costs with and without cirrhosis were $113,568 and $47,436, respectively.

**Conclusion:**

Data from two large healthcare claims databases showed that the majority of patients who had at least one acute-care event experienced additional acute-care events, particularly among those with cirrhosis. Timely treatment to avoid hospitalization and disease progression may help mitigate the clinical and economic burden for patients with PBC.

## Plain language summary: illness burden & costs with primary biliary cholangitis

**What is this article about?:**

Primary biliary cholangitis (PBC) is a chronic liver disease that can worsen over time. Without effective treatment, PBC can cause serious health problems, including reduced quality of life, liver scarring (called cirrhosis), liver failure and death. People with PBC and cirrhosis tend to have other medical conditions and use more healthcare services than people without cirrhosis. In using two large national claims datasets, this study looked at how often healthcare services, especially hospitalizations and emergency department (ED) visits, are used and how much these services cost on an average for patients with PBC, with and without cirrhosis.

**What were the results?:**

One in five patients diagnosed with PBC had cirrhosis, and patients with cirrhosis had more concomitant medical conditions. More than half of patients with cirrhosis who had at least one hospitalization or ED visit for any reason had additional acute-care visits (hospitalizations or ED visits) in the same year. For patients who were hospitalized or had ED visits, the average medical costs were more than double for patients with cirrhosis ($113,567.55) compared with those without cirrhosis ($47,435.95).

**What do the results mean?:**

Patients who had one acute-care event (hospitalization or ED visit) had additional acute-care events, especially those who also had cirrhosis. Given the high use of healthcare services for advanced stages of PBC, timely treatment to avoid hospitalization and negative clinical outcomes is important.

## Shareable abstract

Patients with untreated or unmanaged #primarybiliarycholangitis may develop #cirrhosis, which can lead to more hospital and emergency room visits. Early treatment leads to better #healthoutcomes and reduces #healthcarecosts.

Primary biliary cholangitis (PBC) is a chronic, progressive autoimmune liver disease characterized by the gradual destruction of the intrahepatic bile ducts, which can advance to cirrhosis and lead to liver transplant or death [1,2]. The prevalence of PBC in the USA has nearly doubled, from 21.7 per 100,000 individuals in 2006 to 40.9 per 100,000 in 2021 [3,4]. PBC is commonly diagnosed in patients who are between 40 and 60 years of age, with women affected at an estimated ratio of 4:1 compared with men [1,3]. Men are often diagnosed later and with more advanced stages of the disease compared with women [1]. Patients are generally ‘asymptomatic’ at the time of PBC diagnosis, but most will develop symptoms and signs of cirrhosis and possibly liver failure within 20 years [1]. A more severe, progressive form of PBC can result in the early development of liver fibrosis and liver failure, affecting about 30% of patients, especially men [5]. Ursodeoxycholic acid (UDCA) is approved for first-line therapy for PBC [6]. For patients with PBC who have an inadequate response to UDCA or are intolerant of UDCA, available second-line treatment options include obeticholic acid, elafibranor and seladelpar [7–9].

Patients with PBC tend to have a high comorbidity burden [10], healthcare resource utilization (HCRU) [11] and cost burden [12,13]. Due to the chronic, progressive nature of PBC, the development of cirrhosis has been linked to further negative clinical outcomes [5,14]. A real-world analysis of administrative claims data reported the total and PBC-related costs and high comorbidity burden; this analysis included a sample of 5157 patients, and almost two-thirds of patients were 65 years of age or older [10]. Younossi and colleagues have reported that among patients with PBC, those with cirrhosis have a greater number of hospitalizations, emergency department (ED) visits, and outpatient visits than patients without cirrhosis; the associated costs were not included in the study [11]. Other economic-related studies have focused on hospitalized patients with PBC (without differentiation of cirrhosis diagnosis) and in specific populations, namely Medicare [12,13]. Given the lack of comprehensive assessment of HCRU and costs for acute care (hospitalizations and ED visits) in the PBC population irrespective of payer, we conducted an observational study in two separate administrative claims data sources. The objective of this observational study was to assess general patient characteristics, HCRU (hospitalizations, ED visits, outpatient visits) and prevalence of multiple hospitalizations for patients with PBC with or without cirrhosis using the Komodo’s Healthcare Map™ (Komodo Health) and Optum Clinformatics^®^ Data Mart (CDM) database. The costs of acute care (hospital and ED visits) were derived from the Optum CDM database.

## Materials & methods

### Study design & data sources

**Figure 1.**
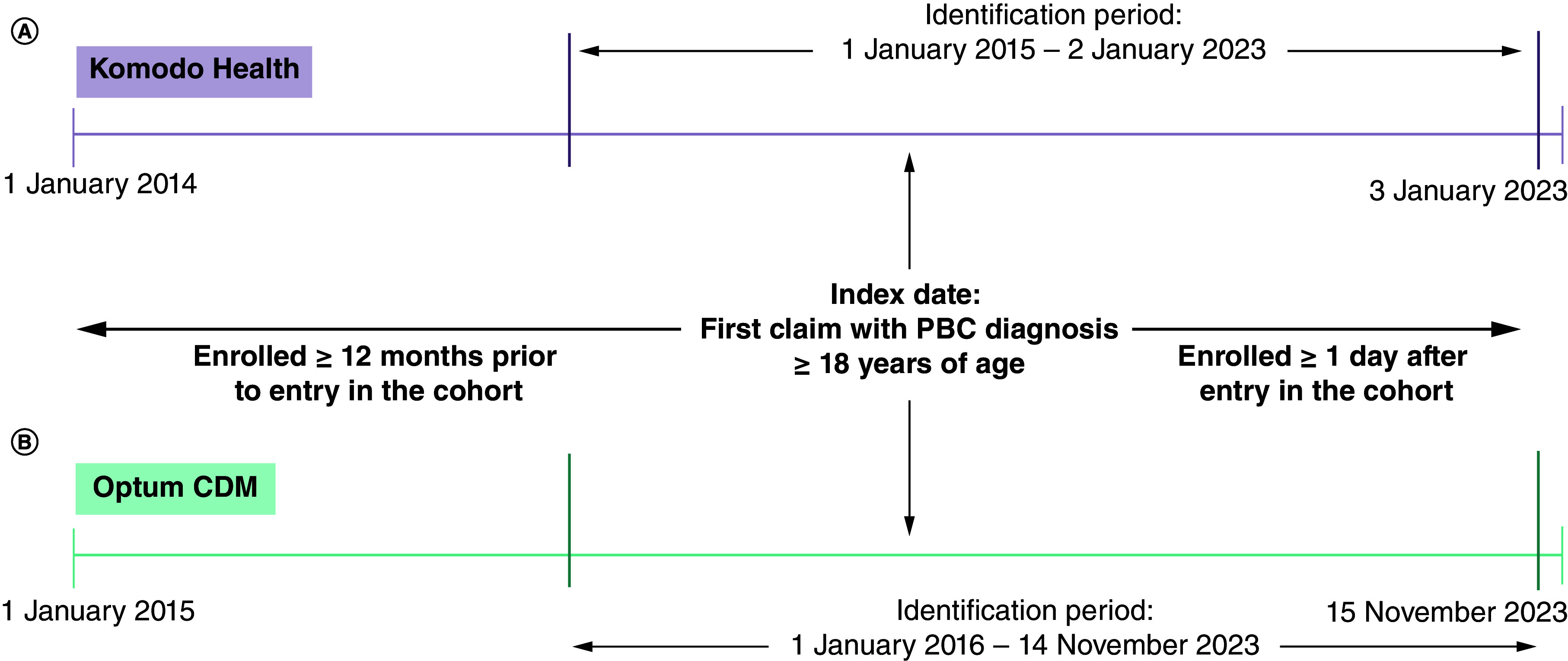
Timeline of the retrospective observational cohort study using two datasets. The two administrative claims data source include **(A)** Komodo’s Healthcare Map™ administrative claims database and **(B)** Optum Clinformatics^®^ Data Mart database. CDM: Clinformatics Data Mart; PBC: Primary biliary cholangitis.

This retrospective observational cohort study was conducted using two datasets (Komodo Health and Optum CDM) that contain deidentified inpatient, outpatient, and pharmacy claims from patients in the US (Figure 1). Komodo Health is a nationally representative, longitudinal claims database that sources claims data from more than 330 million patients covered by commercial, Medicare, and Medicaid plans [15,16]. In Komodo Health, Datavant^®^ tokenization methodology was used to allow patient records to be matched across health plans without sharing protected health information [17]. Optum CDM contains claims data derived from 81 million patients across all 50 US states who are covered by commercial and Medicare plans [18]. Patient characteristics and HCRU were assessed in both datasets. However, due to missing cost data in the Komodo Health data (>40%) available to the researchers, annualized medical (i.e., inpatient and outpatient) costs were calculated with Optum CDM data only.

### Study population

**Figure 2.**
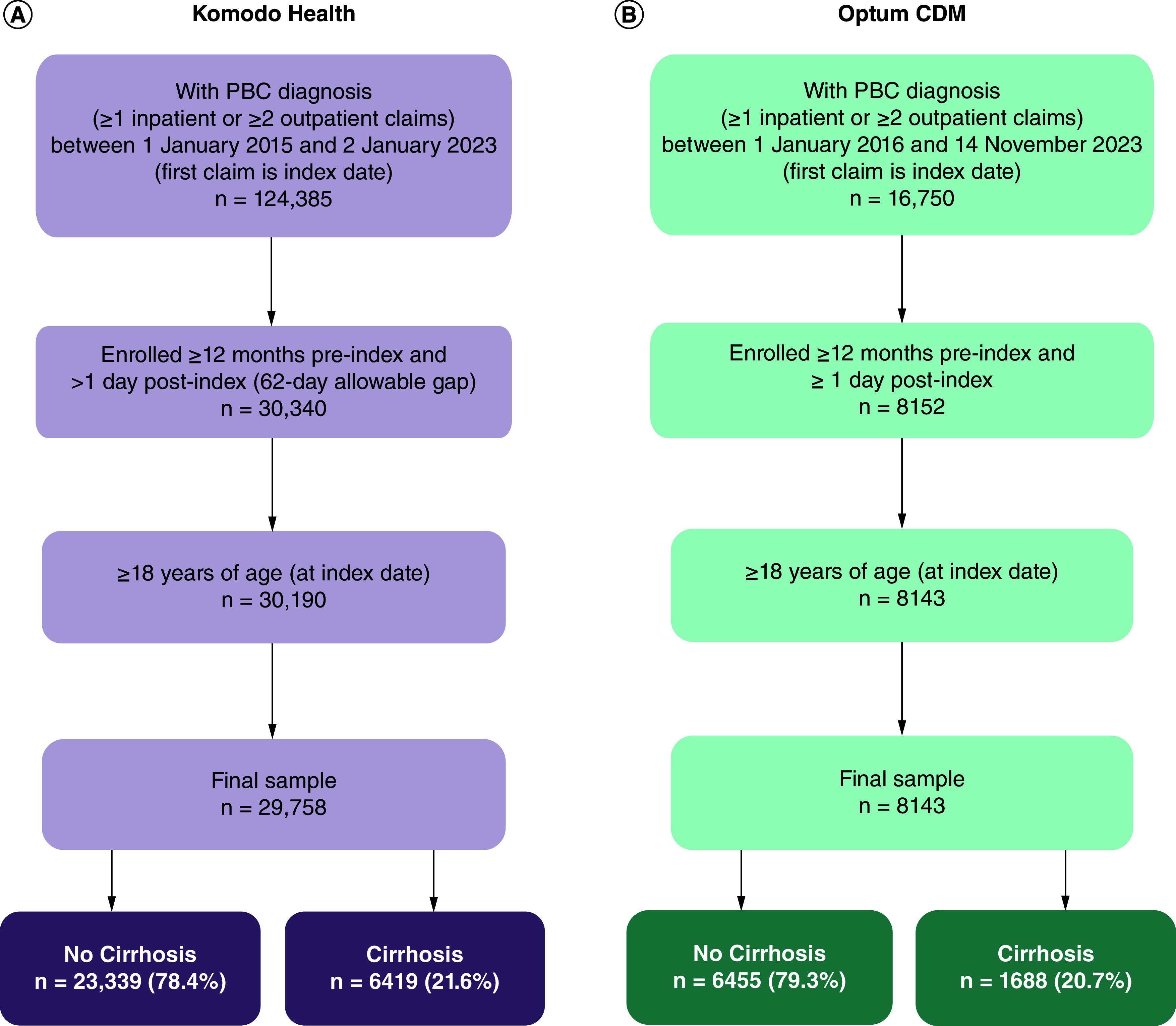
Main patient selection criteria used in the study. The two databases included similar selection criteria from **(A)** Komodo’s Healthcare Map™ administrative claims database and **(B)** Optum Clinformatics^®^ Data Mart database. CDM: Clinformatics Data Mart; ICD: International Classification of Diseases; PBC: Primary biliary cholangitis.

Patients with a diagnosis of PBC based on PBC diagnosis codes in any position in ≥1 inpatient claim or ≥2 outpatient claims on different days were identified from Komodo Health (between 1 January 2015 and 2 January 2023) and Optum CDM (between 1 January 2016 and 14 November 2023) (Figure 2). The International Classification of Diseases (ICD)-9 code 571.6 and ICD-10 code K74.3 were used for PBC diagnosis based on previous studies that have validated the ICD codes in PBC and the algorithm using administrative data [19,20]. The date of the first claim with a PBC diagnosis code during the identification period was set as the index date or date of entry into the cohort. Patients were ≥18 years of age at the index date and enrolled in a health plan for ≥12 months pre-index and ≥1 day post-index (62-day allowable gap in continuous enrollment). Patients were required to be enrolled in a health plan for ≥12 months pre-index, allowing adequate time to capture comorbid conditions. Patients were assessed for the presence of cirrhosis during the 12 months prior to entry into the cohort and were flagged and assigned to the cirrhosis group based on the presence of ICD-9 or ICD-10 diagnosis codes for cirrhosis [20,21] and a claim for an imaging procedure (using current procedural terminology codes and ICD-9 and ICD-10 procedure codes) within 6 months prior to the cirrhosis diagnosis based on AASLD guidelines for follow-up imaging that is recommended every 6 months for patients with cirrhosis [22] (a full list of diagnosis and procedures codes is provided in Supplementary Table 1).

### Pre-index patient characteristics & comorbidity profile

Demographics, including age, sex, race/ethnicity and type of health insurance plan were obtained at the time of entry into the cohort (12 months pre-index). Pre-index clinical characteristics, including UDCA use, comorbidity profile using the Charlson Comorbidity Index (CCI) [23] and presence of additional autoimmune conditions were assessed. The additional autoimmune conditions assessed included autoimmune hepatitis, autoimmune thyroiditis, celiac disease, polymyositis, Raynaud’s disease, rheumatoid arthritis, scleroderma, Sjögren syndrome, systemic lupus erythematosus and vasculitis.

### Post-index healthcare utilization & cost variables

Measures of HCRU included outpatient visits and acute-care events consisting of hospitalizations and ED visits that did not lead to hospitalization. Overall outpatient visits and outpatient specialist visits were assessed using ‘gastroenterologist’, ‘hepatology/liver medicine’ and ‘endocrinology, diabetes and metabolism’ as search terms in Komodo Health and ‘diabetes, metabolism and endocrinology’, ‘gastroenterology’, ‘hepatology’, ‘transplant hepatology physician’ and ‘toxicology’ in Optum CDM.

Liver-related and pancreatobiliary-related events were identified based on diagnosis codes (ICD-9 or -10) present in the primary diagnosis position. Liver-related hospitalizations and ED visits were identified based on presence of diagnosis codes for alcohol-associated liver disease, toxic liver disease, inflammatory liver disease, chronic and viral hepatitis, hepatic failure, hepatic fibrosis, cirrhosis, hepatic decompensation, liver neoplasms, liver infections, liver complications, liver injury and other liver diseases. Hospitalizations and ED visits for pancreatobiliary-related events, a subset of liver-related events, included cholelithiasis, cholecystitis, acute pancreatitis, pancreatitis-related disorders (e.g., chronic pancreatitis and pancreatic cyst) and biliary duct- and gallbladder-related injury and diseases.

Due to the lack of comprehensive cost information for all identified patients in the Komodo data available to the researchers, annualized overall and acute-care (hospitalizations and ED visits) medical costs were assessed only in the Optum CDM cohort. Liver-related and pancreatobiliary-related events and their associated costs were identified based on diagnosis codes present in the primary diagnosis position. Costs were adjusted to 2023 USD using the US Bureau of Labor Statistics Consumer Price Index for medical care.

### Statistical analyses

Descriptive analyses were used to assess patient characteristics (demographics and clinical characteristics). HCRU parameters were categorized by overall, liver-related and pancreatobiliary-related hospitalizations and ED visits. The proportion of patients with a hospitalization or ED visit who had additional hospitalizations or ED visits was also quantified. All results were reported for the overall population and stratified by the presence or absence of cirrhosis during the 12-month pre-index period. Annualized number of hospitalizations and ED visits were calculated by dividing the total number of hospitalizations and ED visits during the follow-up period by the length of the follow-up period (measured in years). Analyses were stratified by commercial or Medicare insurance enrollment for acute-care events (hospitalizations and ED visits). Insurance was categorized using the hierarchy Medicare > Medicaid > commercial > other, as patients could have multiple types of insurance. The primary insurance coverage associated with a patient’s closed enrollment in which their index date fell was used. If the index date did not fall in a closed enrollment period (due to the 62-day allowable gap), the insurance associated with the first closed enrollment period starting after the index date was used. Annualized medical costs were calculated by dividing the total medical costs during the follow-up period by the length of the follow-up period (measured in years).

## Results

### Patient demographics & clinical characteristics

#### Komodo Health

Overall, 29,758 patients met the inclusion criteria, and 21.6% of patients had cirrhosis (Table 1). A total of 1773 patients with cirrhosis had an ICD-9 diagnosis code, and 4437 patients had an ICD-10 diagnosis code. Among the patients with cirrhosis, 50.4% had Medicaid or Medicare coverage. There was a larger proportion of men in the cirrhosis group compared with the no-cirrhosis group. The mean (SD) age of the overall population was 59.2 (13.2) years and was consistent across the subgroups of patients with or without cirrhosis. The overall mean (SD) CCI was 3.1 (2.5), and a higher percentage of patients with cirrhosis at the time of study entry had a CCI >1 compared with patients without cirrhosis.

**Table 1.** Demographic and clinical characteristics for patients with primary biliary cholangitis with and without cirrhosis.

	Komodo Health^†^^,^^‡^			Optum CDM^†^^,^^‡^		
	Overall (n = 29,758)	Cirrhosis (n = 6419)	No cirrhosis (n = 23,339)	Overall (n = 8143)	Cirrhosis (n = 1688)	No cirrhosis (n = 6455)
Demographics						
**Sex, n (%)**						
Female	23,908 (80.3)	4381 (68.3)	19,527 (83.7)	6578 (80.8)	1186 (70.3)	5392 (83.5)
Male	5850 (19.7)	2038 (31.7)	3812 (16.3)	1565 (19.2)	502 (29.7)	1063 (16.5)
**Age at index, years**						
Mean (SD)	59.2 (13.2)	59.3 (13.0)	59.2 (13.2)	67.0 (12.7)	67.3 (12.4)	67.0 (12.8)
Median (IQR)	60.0 (51.0, 68.0)	60.0 (52.0, 68.0)	60.0 (51.0, 68.0)	69.0 (60.0, 76.0)	69.0 (61.0, 76.0)	69.0 (60.0, 76.0)
**Insurance at index, n (%)**						
Commercial	11,707 (39.3)	2024 (31.5)	9683 (41.5)	2461 (30.2)	436 (25.8)	2025 (31.4)
Medicaid	5107 (17.2)	1598 (24.9)	3509 (15.0)	–	–	–
Medicare	7257 (24.4)	1639 (25.5)	5618 (24.1)	5678 (69.7)	1252 (74.2)	4426 (68.6)
Other^§^	5687 (19.1)	4529 (19.4)	1158 (18.0)	4 (<0.1)	0	4 (0.1)
**Race/ethnicity, n (%)**						
Asian	828 (2.8)	171 (2.7%)	657 (2.8)	222 (2.7)	30 (1.8)	192 (3.0)
Black or African–American	2028 (6.8)	574 (8.9)	1454 (6.2)	693 (8.5)	148 (8.8)	545 (8.4)
Hispanic or Latino	4483 (15.1)	1187 (18.5)	3296 (14.1)	1097 (13.5)	267 (15.8)	830 (12.9)
White	15,385 (51.7)	3208 (50.0)	12,177 (52.2)	5651 (69.4)	1129 (66.9)	4522 (70.1)
Other	921 (3.1)	197 (3.1%)	724 (3.1)	–	–	–
Missing	–	–	–	382 (4.7)	86 (5.1)	296 (4.6)
Unknown	6113 (20.5)	1082 (16.9%)	5031 (21.6)	98 (1.2)	28 (1.7)	70 (1.1)
**Clinical characteristics**						
**UDCA, n (%)**	14,470 (48.6)	2989 (46.6)	11,481 (49.2)	3536 (43.4)	728 (43.1)	2808 (43.5)
**CCI, n (%)**						
Mean (SD)	3.1 (2.5)	4.8 (2.9)	2.6 (2.1)	3.6 (2.7)	5.2 (3.1)	3.2 (2.5)
Median (IQR)	2.0 (1.0, 4.0)	4.0 (3.0, 6.0)	2.0 (1.0, 3.0)	3.0 (1.0, 5.0)	5.0 (3.0, 7.0)	2.0 (1.0, 4.0)
CCI >1	19,483 (65.5)	5614 (87.5)	13,869 (59.4)	5936 (72.9)	1505 (89.2)	4431 (68.6)
**CCI components occurring in >10% of patients in either Komodo Health or Optum CDM**						
Diabetes without chronic complication	7729 (26.0)	2486 (38.7)	5243 (22.5)	2301 (28.3)	648 (38.4)	1653 (25.6)
Chronic pulmonary disease	6798 (22.8)	1906 (29.7)	4892 (21.0)	2057 (25.3)	521 (30.9)	1536 (23.8)
Liver disease^¶^ (moderate to severe)	5270 (17.7)	3725 (58.0)	1545 (6.6)	1510 (18.5)	985 (58.4)	525 (8.1)
Renal disease	3770 (12.7)	1389 (21.6)	2381 (10.2)	1621 (19.9)	518 (30.7)	1103 (17.1)
Peripheral vascular disease	3592 (12.1)	1163 (18.1)	2429 (10.4)	1596 (19.6)	437 (25.9)	1159 (18.0)
Rheumatic disease/connective tissue disease	3457 (11.6)	660 (10.3)	2797 (12.0)	978 (12.0)	175 (10.4)	803 (12.4)
Malignancy (any cancer)	3396 (11.4)	1072 (16.7)	2324 (10.0)	1116 (13.7)^#^	302 (17.9)	814 (12.6)
Diabetes with chronic complication	3224 (10.8)	1223 (19.1)	2001 (8.6)	1245 (15.3)	405 (24.0)	840 (13.0)
Congestive heart failure	2876 (9.7)	1189 (18.5)	1687 (7.2)	1113 (13.7)	372 (22.0)	741 (11.5)
Cerebrovascular disease	2577 (8.7)	806 (12.6)	1771 (7.6)	1029 (12.6)	255 (15.1)	774 (12.0)
**Diagnosed with an autoimmune disease (in addition to PBC), n (%)**	7406 (24.9)	1784 (27.8)	5622 (24.1)	1810 (22.2)	442 (26.2)	1368 (21.2)

† Cirrhosis and no cirrhosis were identified at the time of entry in the cohort.

‡ Patients flagged with cirrhosis based on diagnosis code (ICD-9 or -10) and the claim for imaging procedure within 6 months of the cirrhosis of diagnosis.

§ Patients with multiple types of insurance.

¶ Liver disease includes esophageal varices with or without bleeding, portal hypertension, hepatic encephalopathy, hepatorenal syndrome, gastric varices, alcoholic liver failure, hepatic failure, chronic liver failure, toxic liver disease with hepatic necrosis and hepatic veno-occlusive disease.

# Any malignancy, including lymphoma and leukemia, except malignant neoplasm of the skin.

CCI: Charlson Comorbidity Index; CDM: Clinformatics Data Mart; ICD: International Classification of Diseases; PBC: Primary biliary cholangitis; UDCA: Ursodeoxycholic acid.

#### Optum CDM

Of the 8143 patients identified with PBC, 20.7% of patients had cirrhosis (Table 1). A total of 92 patients with cirrhosis had an ICD-9 diagnosis code, and 1454 had an ICD-10 diagnosis code. The cirrhosis group had a larger proportion of men compared with the no-cirrhosis group. The mean (SD) age of the subgroups of patients with or without cirrhosis was consistent with the overall population (67.0 [12.7] years). The majority of patients were enrolled in Medicare (69.7%). The overall mean (SD) CCI was 3.6 (2.7), and a higher percentage of patients with cirrhosis at the time of study entry had a CCI >1 compared with patients without cirrhosis.

### Annualized hospitalizations, ED visits & outpatient visits

#### Komodo Health

Annually, 20.8% of patients had at least one hospitalization (Table 2). Among these patients, 60.2% had at least one additional hospitalization. A higher proportion of patients with cirrhosis had at least one hospitalization compared with patients without cirrhosis. The mean (SD) length of hospital stay (days) was longer in the cirrhosis cohort versus no-cirrhosis cohort. Among patients who had at least one liver-related or pancreatobiliary-related hospitalization, 60.1 and 48.5%, respectively, had at least one additional hospitalization. Overall, 11.4% of patients had at least one ED visit that did not lead to hospitalization, and among these patients, 44.6% had at least one additional ED visit. The greatest proportion of patients with at least one hospitalization had Medicaid coverage (31.2%; n = 5107), followed by Medicare (26.0%; n = 7257), and commercial insurance (14.4%; n = 11,707) (Table 3). Among patients with Medicaid coverage, 25% had at least one ED visit that did not lead to hospitalization.

**Table 2.** Healthcare resource utilization in patients with primary biliary cholangitis with and without cirrhosis.

Annualized HCRU^†^^,^^‡^	Komodo Health^§^			Optum CDM^§^		
	Overall (n = 29,758)	Cirrhosis^¶^ (n = 6419)	No cirrhosis (n = 23,339)	Overall (n = 8143)	Cirrhosis^¶^ (n = 1688)	No cirrhosis (n = 6455)
**Hospitalizations, n (%)**						
≥1 hospitalization	6158 (20.8)	2668 (41.6)	3517 (15.1)	1612 (19.8)	617 (36.6)	995 (15.4)
Additional hospitalizations among those with a hospitalization^#^	3726 (60.2)	1849 (69.3)	1877 (53.4)	1006 (62.4)	443 (71.8)	563 (56.6)
**Liver-related hospitalizations^††^, n (%)**						
≥1 hospitalization	2993 (10.1)	1838 (28.6)	1155 (4.9)	422 (5.2)	254 (15.0)	168 (2.6)
Additional hospitalizations among those with a hospitalization^‡‡^	1799 (60.1)	1203 (65.5)	596 (51.6)	251 (59.5)	164 (64.6)	87 (51.8)
**Pancreatobiliary-related hospitalizations^††^, n (%)**						
≥1 hospitalization	1088 (3.7)	580 (9.0)	508 (2.2)	109 (1.3)	46 (2.7)	63 (1.0)
Additional hospitalizations among those with a hospitalization^#^	528 (48.5)	297 (51.2)	231 (45.5)	57 (52.3)	27 (58.7)	30 (47.7)
**ED visits^††^^,^^‡‡^**, n (%)						
≥1 ED visit	3383 (11.4)	1209 (18.8)	2174 (9.3)	1594 (19.6)	452 (26.8)	1142 (17.7)
Additional ED visits among those with an ED visit^#^	1508 (44.6)	639 (52.9)	869 (40.0)	806 (50.6)	272 (60.1)	534 (46.8)
**Liver-related ED visits^††^^,^^‡‡^, n (%)**						
≥1 ED visit	356 (1.2)	247 (3.8)	109 (0.5)	143 (1.8)	95 (5.6)	48 (0.7)
Additional ED visits among those with an ED visit^#^	175 (49.2)	133 (53.8)	42 (38.5)	75 (52.4)	55 (57.9)	20 (41.7)
**Pancreatobiliary-related ED visits^††^^,^^‡‡^^,^^§§^, n (%)**						
≥1 ED visit	62 (0.2)	30 (0.5)	32 (0.1)	23 (0.3)	12 (0.7)	11 (0.2)
Additional ED visits among those with an ED visit^#^	17 (27.4)	11 (36.7)	6 (18.8)	9 (39.1)	6 (60.0)	3 (27.3)
**Hospitalizations, all patients (n), PPPY**						
Mean (SD)	1.0 (3.1)	2.3 (4.9)	0.7 (2.3)	1.0 (2.8)	2.1 (4.4)	0.7 (2.11)
Median (IQR)	0 (0, 0.7)	0.6 (0, 2.5)	0 (0, 0.4)	0 (0, 0.7)	0.4 (0, 2.1)	0 (0, 0.5)
**Hospitalizations, n, among patients with ≥1 hospitalization, PPPY**						
Mean (SD)	4.4 (5.7)	5.4 (6.4)	3.7 (5.0)	4.4 (5.0)	5.5 (6.0)	3.8 (4.2)
Median (IQR)	2.5 (1.5, 4.9)	3.1 (1.7, 6.5)	2.1 (1.4, 4.0)	2.6 (1.5, 5.2)	3.3 (1.8, 7.0)	2.3 (1.5, 4.3)
**Outpatient visits, n**						
Mean (SD)	21.6 (20.4)	28.1 (27.3)	19.8 (17.6)	30.7 (28.8)	39.7 (35.4)	28.3 (26.3)
Median (IQR)	16.6 (9.2, 28.1)	21.8 (12.4, 36.8)	15.4 (8.6, 25.9)	22.4 (13.1, 38.5)	30.3 (16.7, 51.6)	21.0 (12.6, 35.4)
**Outpatient specialist visits, n** ^¶¶^						
Mean (SD)	2.3 (4.1)	3.7 (6.8)	1.9 (2.8)	2.1 (4.5)	3.1 (6.6)	1.9 (3.7)
Median (IQR)	1.3 (0, 3.0)	2.2 (0.4, 4.9)	1.1 (0, 2.6)	1.1 (0, 2.6)	1.6 (0, 3.7)	1.0 (0. 2.4)
**Length of hospital stay (days), mean (SD), visit level**	4.9 (10.2)	5.6 (11.4)	4.4 (9.3)	9.1 (11.9)	9.1 (11.8)	9.1 (11.9)

† Hospitalization, ED and outpatient visit data are annualized.

‡ Annualized number of hospitalizations and ED visits were calculated by dividing the total number of hospitalizations and ED visits during the follow-up period by the length of the follow-up period (measured in years).

§ Cirrhosis and no cirrhosis were identified at the time of entry in the cohort.

¶ Patients flagged with cirrhosis based on diagnosis code (ICD-9 or -10) and the claim for imaging procedure within 6 months of the cirrhosis of diagnosis.

# Additional hospitalizations or ED visits are among patients with ≥1 hospitalization or ED visit.

†† Based on diagnosis codes in the primary diagnosis position.

‡‡ ED visits that did not lead to a hospitalization.

§§ A subset of liver-related care was reported as pancreatobiliary-related care and included PBC, cholelithiasis, cholecystitis, pancreatic diseases, and biliary duct– and gallbladder-related injuries and diseases.

¶¶ Outpatient specialist visits are with a gastroenterologist, endocrinologist, or hepatologist.

CDM: Clinformatics Data Mart; ED: Emergency department; HCRU: Healthcare resource utilization; ICD: International Classification of Diseases; IQR: Interquartile range; PBC: Primary biliary cholangitis; PPPY: Per patient per year.

**Table 3.** Healthcare resource utilization by insurance coverage in patients with primary biliary cholangitis.

Annualized HCRU^†^^,^^‡^	Komodo Health					Optum CDM			
	Overall (n = 29,758)	Commercial (n = 11,707)	Medicare (n = 7257)	Medicaid (n = 5107)	Other (n = 5687^#^)	Overall (n = 8143)	Commercial (n = 2461)	Medicare (n = 5678)	Other (n = 4^#^)
**Hospitalizations, n (%)**									
≥1 hospitalization	6185 (20.8)	1683 (14.4)	1886 (26.0)	1593 (31.2)	1023 (18.0)	3442 (42.3)	645 (26.2)	2795 (49.2)	2 (50.0)
Additional hospitalizations among those with a hospitalization^§^	3726 (60.2)	967 (57.5)	1102 (58.4)	1052 (20.6)	605 (59.1)	2167 (62.9)	343 (53.2)	1823 (65.2)	1 (25.0)
**ED visits^¶^, n (%)**									
≥1 ED visit	3383 (11.4)	770 (6.6)	811 (11.2)	1276 (25.0)	526 (9.2)	3733 (45.8)	626 (25.4)	3103 (54.6)	4 (100.0)
Additional ED visits among those with an ED visit^§^	1508 (44.6)	275 (35.7)	335 (41.3)	688 (53.9)	210 (39.9)	2405 (64.4)	433 (69.2)	1970 (63.5)	2 (50.0)

† Hospitalization and ED visit data are annualized.

‡ Annualized number of hospitalizations and ED visits were calculated by dividing the total number of hospitalizations and ED visits during the follow-up period by the length of the follow-up period (measured in years).

§ Additional hospitalizations or ED visits are among patients with ≥1 hospitalization or ED visit.

¶ ED visits that did not lead to a hospitalization.

# Patients with multiple types of insurance.

CDM: Clinformatics Data Mart; ED: Emergency department; HCRU: Healthcare resource utilization; PBC: Primary biliary cholangitis.

#### Optum CDM

Annually, 19.8% of patients had at least one hospitalization. Among these patients, 62.4% had at least one additional hospitalization (Table 2). Similar to Komodo Health, a higher proportion of patients with cirrhosis had at least one hospitalization compared with patients without cirrhosis. The mean (SD) length of hospitalization stay (days) was similar in the cirrhosis cohort (9.1 [11.8]) versus no-cirrhosis cohort (9.1 [11.9]). Among patients who had at least one liver-related and pancreatobiliary-related hospitalization, 59.5% and 52.3% had at least one additional hospitalization, respectively. Annually, 19.6% of patients had at least one ED visit; among these patients, 50.6% had at least one additional ED visit. Among patients with Medicare coverage (n = 5678), almost half of the patients had at least one hospitalization (Table 3). Among patients with commercial insurance coverage (n = 2461), 26.2% had at least one hospitalization. For patients who had at least one ED visit that did not lead to hospitalization, 54.6% were covered by Medicare and 25.4% were covered by commercial insurance.

### Annualized medical costs (Optum CDM)

The mean (SD) annual total overall medical cost per patient was $62,149.47 ($173,669.94). The overall, liver-related, and pancreatobiliary-related costs were substantially higher for patients with cirrhosis compared with those without cirrhosis (Table 4). For patients with acute-care events, the overall mean (SD) annual acute-care cost (hospitalizations and ED visits) was $66,598.49 ($182,447.54).

**Table 4.** Annualized medical costs in patients with primary biliary cholangitis (Optum Clinformatics Data Mart)^†^^,^^‡^.

	Medical costs, mean (SD)			Acute-care medical costs (among those with ≥1 acute care event)^§^, mean (SD)		
	Overall (n = 8143)	Cirrhosis (n = 1688)	No cirrhosis (n = 6455)	Overall (n = 8143)	Cirrhosis (n = 1688)	No cirrhosis (n = 6455)
**Overall**	$62,149.47 ($173,669.94)	$124,788.11 ($272,819.42)	$45,769.30 ($131,529.21)	$66,598.49 ($182,447.54)	$113,567.55 ($257,696.89)	$47,435.95 ($136,125.49)
Liver-related	$15,160.74 ($109,762.28)	$48,482.84 ($202,459.27)	$6,446.92 ($64,173.01)	$75,628.59 ($251,560.80)	$104,766.16 ($298,329.33)	$43,752.79 ($182,608.66)
Pancreatobiliary-related^¶^	$3797.81 ($48,373.78)	$7754.02 ($55,725.93)	$2763.24 ($46,207.85)	$47,387.85 ($216,360.01)	$51,483.52 ($141,864.87)	$44,498.01 ($256,552.78)

† Adjusted to 2023 dollars.

‡ Annualized medical costs were calculated by dividing the total medical costs during the follow-up period by the length of the follow-up period (measured in years).

§ Acute care includes hospitalization or ED visit.

¶ A subset of liver-related care was reported as pancreatobiliary-related care and included PBC, cholelithiasis, cholecystitis, pancreatic diseases, and biliary duct– and gallbladder-related injuries and diseases.

CDM: Clinformatics Data Mart; PBC: Primary biliary cholangitis.

## Discussion

This retrospective observational study of patients with PBC, using two large healthcare claims datasets, demonstrated that patients with PBC generally had a high comorbidity burden and HCRU, especially among patients with cirrhosis. A greater proportion of patients with cirrhosis had more than one hospitalization than patients without cirrhosis in both the Komodo Health (41.6 vs 15.1%) and Optum CDM (36.6 vs 15.4%) datasets. Younossi and colleagues reported a mean (SD) number of all-cause inpatient stays of 1.77 (6.18) in all patients with PBC and 3.21 (8.45) specifically in patients with cirrhosis for over 1 year [11]. These were slightly lower in the current study, with an annualized mean (SD) number of all-cause hospitalizations of 1.0 (3.1) in Komodo Health and 1.0 (2.8) in Optum CDM per patient per year. Among patients with cirrhosis, the mean number of hospitalizations was 2.3 (4.9) in Komodo Health and 2.1 (4.4) in Optum CDM. In the current study, among patients with at least one hospitalization, approximately 60% had additional hospitalizations.

Delays in diagnosis and treatment have been associated with a higher risk for disease progression and are noted to contribute to increased morbidity, mortality, and HRCU [5,11]. These delays in diagnosis and treatment may be potentially due to insufficient or lack of access to care and health insurance coverage. In Komodo Health, 17.2% of the sample was enrolled in Medicaid whereas in Optum CDM, approximately 99% of the sample consisted of Medicare and commercial insurance enrollees. In Komodo Health, a higher proportion of patients with Medicaid (31.2%) had at least one hospitalization than those enrolled in Medicare (26.0%) or commercial insurance (14.4%). Similar results were observed for ED visits. These findings suggest that lower-income patients with PBC have a greater burden with respect to hospitalizations and ED visits. In Komodo Health, a higher proportion of patients with cirrhosis had Medicaid coverage, whereas a higher proportion of patients without cirrhosis had commercial insurance, which also highlights a potentially greater PBC burden among low-income patients. In Optum CDM, a higher proportion of patients with Medicare (49.2%) had at least one hospitalization than those enrolled in commercial insurance (26.4%). Additionally, a higher proportion of patients with cirrhosis had Medicare coverage, whereas a higher proportion of those without cirrhosis had commercial insurance. Due to a lack of Medicaid enrollees in Optum CDM, we were unable to assess trends in this population in that dataset.

In studies conducted using the national inpatient sample (NIS), patients with Medicaid insurance had higher odds of in-hospital mortality compared with those with private or commercial insurance [24] or Medicare coverage [13]. Younossi *et al.* reported that patients with PBC enrolled in Medicaid had twice the chance of developing cirrhosis than patients enrolled in a commercial insurance plan [11]. In patients with PBC, disparities in access to healthcare can contribute to delayed diagnosis and treatment, especially for those with Medicaid coverage [24]. A meta-analysis found that, in general, patients with Medicaid had greater difficulty obtaining appointments with primary care physicians and specialty care compared with patients with private insurance [25]. Further studies are needed to examine differences in HCRU for patients with various sociodemographic characteristics.

Although the proportion of men and women in the overall population was consistent with other studies [12,13], the cirrhosis cohort had a larger proportion of men than the other cohorts. In some studies, men with PBC have been reported to experience more complications and have a higher risk for hepatic decompensation and mortality than women with PBC [12,26]. This may be explained by delayed diagnosis of PBC among men and/or more aggressive PBC disease in men.

The HCRU and cost burden for patients with PBC is highlighted by the fact that most patients who experienced an acute-care event (i.e., hospitalization or ED visit) experienced at least one additional acute-care event. This is a key finding of this study, which emphasizes the importance of monitoring PBC patients regularly, with the goal of delaying disease progression and preventing hospitalizations and ED visits. Among patients with at least one acute-care event, the average overall and liver-related medical costs per year were similar, suggesting that most of the acute-care costs might have been attributed to liver-related events. Our findings of a mean (SD) total annualized medical costs (2023 USD) for acute care $66,598.49 ($182,447.54) are comparable to other studies. In a study using a Medicare fee-for-service 5% random sample of hospitalized patients with PBC, the average charge per hospitalization was $72,233 between 2005 and 2015 [12]. In a study conducted using NIS data from 2007 to 2014, the average charge per hospitalization was $73,093 in 2014 [27]. In a similar study conducted with NIS data from 2005 to 2014, the average total charge per hospital discharge was $57,613 in 2014 [13]. While these charges calculated for 2014 in two different studies were different, it should be noted there were differences in selection of patients that may have contributed to the differences in the hospitalization charges observed.

For patients with acute-care events, the annualized mean medical costs were more than double for patients with cirrhosis ($113,567.55) compared with those without cirrhosis ($47,435.95). This finding may be attributed to higher HCRU, particularly as a greater number of hospitalizations and ED visits were observed among patients with cirrhosis. Overall, these findings point to the increased economic burden among patients with advanced disease states. Of note, among patients with at least one acute-care event, the annualized pancreatobiliary-related medical costs per patient with and without cirrhosis were similar. This may be due to the reasons for hospitalization even though more patients in the cirrhosis group had pancreatobiliary-related hospitalizations than the no-cirrhosis group.

Some differences were observed between the two datasets, including the higher mean age (67 vs 59 years), higher proportion of White patients (69.4 vs 51.7%), and more individuals enrolled in Medicare (69.7 vs 24.4%) in Optum CDM than Komodo Health. Furthermore, patients in Optum CDM had a slightly higher CCI (mean: 3.6 vs 3.1) than patients from Komodo Health. Higher mean specialist outpatient visits were observed for patients with cirrhosis in Komodo Health than Optum CDM (3.7 vs 3.1). The duration of hospital stay was longer for patients in Optum CDM than for those in Komodo Health (9 vs 5 days), likely due to the older mean age of the Optum CDM sample. We observed some differences in the results for the two datasets for the length of stay that was evaluated by presence or absence of cirrhosis. In Komodo Health, the duration of stay for hospitalizations was longer in patients with cirrhosis compared with those without cirrhosis, which might contribute to high medical costs. However, in Optum CDM, although the length of stay was longer than that observed in Komodo Health, there were no differences in the average length of hospital stay between patients with cirrhosis and those without cirrhosis.

PBC disease progression and the higher comorbidity burden for patients with cirrhosis versus those without cirrhosis may contribute to increased HCRU and, consequently, increased costs. Patients with PBC had significant comorbidity burden (e.g., diabetes, chronic obstructive pulmonary disease and liver disease), and more than two-thirds of patients had a CCI >1 at the time of study entry. Moreover, patients with cirrhosis had a CCI of approximately 5 in both databases, which can lead to a high clinical and economic burden. In addition to the direct (HCRU-related) costs, the indirect costs (e.g., absenteeism from work, caregiver burden, fatigue and mental health strains/reduced quality of life) associated with a chronic illness likely posed a heavy burden for patients with PBC, particularly for those with cirrhosis.

### Limitations

There are several limitations to this study, and the results should be interpreted within the context of these limitations. These datasets have large samples of patients and include patients across the country, but they may not represent the entire PBC population across the US. Although claims data provide longitudinal data on patients with PBC who require healthcare services, patient information may be incomplete. Detailed clinical information, including all lab results, date of diagnosis, duration of disease, fibrosis information, other disease-specific information, prescriptions filled beyond the dates the patient was enrolled in the health plan and complete treatment history, were not captured in the dataset. The dataset is limited to the time period during which the patient was enrolled in a specific health plan and included in Komodo Health or Optum CDM; hence, the complete patient history may not be available.

This study focused on assessing HCRU and cost for patients with PBC and included information related to patient encounters during a certain period of time. Accessing patient medical records could offer additional insights into circumstances (e.g., PBC symptoms), stage of the disease, and other information that drive patients to seek additional healthcare services or reasons for particular patient or physician decisions. Some patients or events may have been omitted due to coding errors, and data regarding the HCRU of uninsured patients with PBC were unavailable. Insurance-related analysis was based on coverage at the time of the index and not the entire study period. This study provides insights into HCRU among patients with PBC, particularly acute care among this patient population. While we were able to estimate costs from Optum CDM, it should be noted that deductibles, copays and coinsurance were not considered. Furthermore, the findings from Optum CDM cost data are from a single payer and may not apply to all payers. Nevertheless, the use of two real-world claims datasets provides insight into the type and rates of HCRU in patients with PBC with or without cirrhosis.

## Conclusion

This study provides an overview of the HCRU and costs for patients with PBC. Rates of HCRU among patients with PBC were similar in two large healthcare claims databases. Patients who had an acute-care event (i.e., hospitalization or ED visit) experienced subsequent acute-care events (>50% subsequent hospitalization; >40% subsequent ED visit), especially among those with cirrhosis. Furthermore, findings suggest that patients with PBC and cirrhosis tended to have a higher comorbidity burden and more acute-care events (hospitalizations and/or ED visits) than those without cirrhosis. The findings support the importance of timely treatment to prevent hospitalization and disease progression to mitigate the healthcare burden in patients with PBC.

## Summary points

- Delayed or inadequate treatment of primary biliary cholangitis (PBC) can lead to faster disease progression, higher morbidity and mortality and greater healthcare resource utilization (HCRU).
- The objective of this retrospective observational cohort study was to assess general characteristics and HCRU and associated acute-care (hospitalizations and emergency department [ED] visits) costs for patients with PBC with or without cirrhosis using the Komodo’s Healthcare Map™ (Komodo Health) and Optum Clinformatics^®^ Data Mart (CDM) database.
- About 20% of patients with PBC had cirrhosis, and a higher percentage of patients with cirrhosis had Charlson Comorbidity Index >1 at the time of study entry compared with patients without cirrhosis in both datasets.
- More than half of patients with cirrhosis who had at least one acute-care event (hospitalization or ED visit) experienced additional acute-care events.
- In both datasets, the number of hospitalizations and outpatient visits were greater for patients with cirrhosis compared with those without cirrhosis.
- The duration of stay for hospitalizations was longer in patients with cirrhosis compared with those without cirrhosis in Komodo Health, and the hospital stay duration was longer overall in Optum CDM compared with Komodo Health.
- The overall mean annualized medical cost per patient was $62,149.47, and for patients with acute-care events, this cost was $66,598.49.
- Among patients with at least one acute-care event, the mean annualized medical costs were $113,567.55 for patients with cirrhosis and $47,435.95 for those without cirrhosis.
- Timely and effective treatment to avoid hospitalization and delay disease progression may decrease the economic burden for patients with PBC.

## Supplementary Material


