# Supplementary material for: Burden of illness for patients with primary biliary cholangitis: an observational study of clinical characteristics and healthcare resource utilization
Source: J Comp Eff Res. 2025 Mar 6;14(4):e240174. doi: 10.57264/cer-2024-0174 (PMC11963345; doi:10.57264/cer-2024-0174)
Supplement: Supplementary file 1 [file cer-14-240174-s1.docx]

**Supplementary Table 1.** Codes used for cirrhosis diagnosis and imaging biopsy procedures

| **Cirrhosis diagnosis** | | |
| --- | --- | --- |
| **Code** | **Type** | **Description** |
| K7030 | ICD-10-CM | Alcoholic cirrhosis of liver without ascites |
| K7031 | ICD-10-CM | Alcoholic cirrhosis of liver with ascites |
| K744 | ICD-10-CM | Secondary biliary cirrhosis |
| K745 | ICD-10-CM | Biliary cirrhosis, unspecified |
| K7460 | ICD-10-CM | Unspecified cirrhosis of liver |
| K7469 | ICD-10-CM | Other cirrhosis of liver |
| 5712 | ICD-9-CM | Alcohol cirrhosis of liver |
| 5715 | ICD-9-CM | Cirrhosis of liver, not otherwise specified |
|  | | |
| **Imaging biopsy procedure** | | |
| **Code** | **Type** | **Description** |
| 47000 | CPT | Biopsy of liver, needle; percutaneous |
| 47001 | CPT | Biopsy of liver, needle; when done for indicated purpose at time of other major procedure |
| 47100 | CPT | Biopsy of liver, wedge |
| 91200 | CPT | Other diagnostic gastroenterology procedures |
| 76700 | CPT | Ultrasound, abdominal, real time with image documentation; complete |
| 76705 | CPT | Ultrasound, abdominal, real-time with image documentation; limited |
| 74181 | CPT | MRI of the abdomen without contrast |
| 74182 | CPT | MRI of the abdomen with contrast |
| 74183 | CPT | MRI of the abdomen without contrast, followed by contrast |
| 74185 | CPT | Magnetic resonance angiography, abdomen, with or without contrast |
| 74150 | CPT | CT scan of abdomen without contrast |
| 74160 | CPT | CT scan of abdomen with contrast |
| 74170 | CPT | CT scan without contrast, followed by contrast |
| 0FB00ZX | ICD-10-PCS | Excision of liver, open approach, diagnostic |
| 0FB03ZX | ICD-10-PCS | Excision of liver, percutaneous approach, diagnostic |
| 0FB04ZX | ICD-10-PCS | Excision of liver, percutaneous endoscopic approach, diagnostic |
| 0FB10ZX | ICD-10-PCS | Excision of right lobe liver, open approach, diagnostic |
| 0FB13ZX | ICD-10-PCS | Excision of right lobe liver, percutaneous approach, diagnostic |
| 0FB14ZX | ICD-10-PCS | Excision of right lobe liver, percutaneous endoscopic approach, diagnostic |
| 0FB20ZX | ICD-10-PCS | Excision of left lobe liver, open approach, diagnostic |
| 0FB23ZX | ICD-10-PCS | Excision of left lobe liver, percutaneous approach, diagnostic |
| 0FB24ZX | ICD-10-PCS | Excision of left lobe liver, percutaneous endoscopic approach, diagnostic |
| BF45ZZZ | ICD-10-PCS | Ultrasonography of liver |
| BF46ZZZ | ICD-10-PCS | Ultrasonography of liver and spleen |
| BF35Y0Z | ICD-10-PCS | MRI of liver using other contrast, unenhanced and enhanced |
| BF35YZZ | ICD-10-PCS | MRI of liver using other contrast |
| BF35ZZZ | ICD-10-PCS | MRI of liver |
| BF36Y0Z | ICD-10-PCS | MRI of liver and spleen using other contrast, unenhanced and enhanced |
| BF36YZZ | ICD-10-PCS | MRI of liver and spleen using other contrast |
| BF36ZZZ | ICD-10-PCS | MRI of liver and spleen |
| BF2500Z | ICD-10-PCS | CT scan of liver using high osmolar contrast, unenhanced and enhanced |
| BF250ZZ | ICD-10-PCS | CT scan of liver using high osmolar contrast |
| BF2510Z | ICD-10-PCS | CT scan of liver using low osmolar contrast, unenhanced and enhanced |
| BF251ZZ | ICD-10-PCS | CT scan of liver using low osmolar contrast |
| BF25Y0Z | ICD-10-PCS | CT scan of liver using other contrast, unenhanced and enhanced |
| BF25YZZ | ICD-10-PCS | CT scan of liver using other contrast |
| BF25ZZZ | ICD-10-PCS | CT scan of liver |
| BF2600Z | ICD-10-PCS | CT scan of liver and spleen using high osmolar contrast, unenhanced and enhanced |
| BF260ZZ | ICD-10-PCS | CT scan of liver and spleen using high osmolar contrast |
| BF2610Z | ICD-10-PCS | CT scan of liver and spleen using low osmolar contrast, unenhanced and enhanced |
| BF261ZZ | ICD-10-PCS | CT scan of liver and spleen using low osmolar contrast |
| BF26Y0Z | ICD-10-PCS | CT scan of liver and spleen using other contrast, unenhanced and enhanced |
| BF26YZZ | ICD-10-PCS | CT scan of liver and spleen using other contrast |
| BF26ZZZ | ICD-10-PCS | CT scan of liver and spleen |
| BF2C00Z | ICD-10-PCS | CT scan of hepatobiliary system, all using high osmolar contrast, unenhanced and enhanced |
| BF2C0ZZ | ICD-10-PCS | CT scan of hepatobiliary system, all using high osmolar contrast |
| BF2C10Z | ICD-10-PCS | CT scan of hepatobiliary system, all using low osmolar contrast, unenhanced and enhanced |
| BF2C1ZZ | ICD-10-PCS | CT scan of hepatobiliary system, all using low osmolar contrast |
| BF2CY0Z | ICD-10-PCS | CT scan of hepatobiliary system, all using other contrast, unenhanced and enhanced |
| BF2CYZZ | ICD-10-PCS | CT scan) of hepatobiliary system, all using other contrast |
| BF2CZZZ | ICD-10-PCS | CT scan of hepatobiliary system, all |
| BW2000Z | ICD-10-PCS | CT scan of abdomen using high osmolar contrast, unenhanced and enhanced |
| BW200ZZ | ICD-10-PCS | CT scan of abdomen using high osmolar contrast |
| BW2010Z | ICD-10-PCS | CT scan of abdomen using low osmolar contrast, unenhanced and enhanced |
| BW201ZZ | ICD-10-PCS | CT scan of abdomen using low osmolar contrast |
| BW20Y0Z | ICD-10-PCS | CT scan of abdomen using other contrast, unenhanced and enhanced |
| BW20YZZ | ICD-10-PCS | CT scan of abdomen using other contrast |
| BW20ZZZ | ICD-10-PCS | CT scan of abdomen |
| 5012 | ICD-9-PCS | Open liver biopsy |
| 5011 | ICD-9-PCS | Closed liver biopsy |
| 5014 | ICD-9-PCS | Laparoscopic liver biopsy |
| 8874 | ICD-9-PCS | Diagnostic ultrasound-digestive |
| 8897 | ICD-9-PCS | MRI, unspecified |
| 8801 | ICD-9-PCS | CT scan of abdomen |

Abbreviations: CM, clinical modification; CT, computed tomography; CPT, current procedural terminology; ICD, International Classification of Diseases; MRI, magnetic resonance imaging; PCS, procedure coding system.
